# Potent bisphosphonate therapy for preventing fractures after denosumab discontinuation in osteoporosis: A GRADE-assessed systematic review and meta-analysis

**DOI:** 10.1097/MD.0000000000050542

**Published:** 2026-09-04

**Authors:** Asim Shah, Suleman Khan, Syeda Vilay Zehra Rizvi, Kanza Farhan, Misbah Uddin, Syeda Umme Abiha Rizvi, Sabika Fatima, Ang Raj Karan, Aizaz Anwar Khalid, Ahmad Ali, Somaiya Ahmed

**Affiliations:** a Department of Internal Medicine, Khyber Medical College, Peshawar, Pakistan; b Department of Internal Medicine, Jinnah Sindh Medical University, Karachi, Pakistan; c Department of Internal Medicine, Peshawar Medical College, Peshawar, Pakistan; d Department of Internal Medicine, Sir Salimullah Medical College, Dhaka, Bangladesh.

**Keywords:** alendronate, denosumab discontinuation, meta-analysis, osteoporosis, potent bisphosphonates, sequential therapy, vertebral fractures, zoledronic acid

## Abstract

**Background:**

Denosumab cessation causes rebound bone turnover, fast bone loss, and an elevated risk of vertebral fractures. Pooled data about the fracture prevention efficacy of potent bisphosphonates (BPs) as sequential therapy have been lacking to counteract this risk.

**Methods:**

This systematic review and meta-analysis was carried out according to the Preferred Reporting Items for Systematic Reviews and Meta-Analyses 2020 guidelines. PubMed, Scopus, the Cochrane Library, and ClinicalTrials.gov were searched from the beginning through May 1, 2026. Included studies comprised adults with osteoporosis or low bone mass who discontinued denosumab and subsequently received potent BP therapy, defined as alendronate or zoledronic acid, compared with no subsequent antiresorptive therapy or a selective estrogen receptor modulator (SERM). Random-effects meta-analyses were performed using the Mantel–Haenszel method to calculate pooled risk ratios (RRs) with 95% confidence intervals (CIs). Prespecified subgroup analyses were conducted based on comparator type. The Grading of Recommendations Assessment, Development and Evaluation assessment method was applied to assess the certainty of the evidence.

**Results:**

Six studies were included; 683 participants contributed to the primary incident vertebral fracture analysis. Potent BP therapy was linked to a considerable reduction in incident vertebral fractures (RR = 0.29; 95% CI: 0.14–0.59; 71% relative risk reduction), multiple vertebral fractures (RR = 0.08; 95% CI: 0.01–0.43; 92% relative risk reduction), and clinical vertebral fractures (RR = 0.18; 95% CI: 0.07–0.49; 82% relative risk reduction). The protective effect was driven mainly by comparisons with no subsequent therapy, while comparisons with SERM therapy were limited and not statistically significant. There was no statistically significant reduction in any fracture (RR = 0.35; 95% CI: 0.10–1.24) or non-vertebral fracture (RR = 0.43; 95% CI: 0.07–2.64). Overall certainty of evidence was low to very low, mainly due to the observational nature of most included studies and imprecision in several outcomes.

**Conclusion:**

Potent BP therapy was related to decreased vertebral fracture risk after denosumab termination, particularly when compared with no subsequent therapy. Evidence versus SERM comparators was limited and inconclusive. The results are in agreement with the present clinical guideline recommendations for sequential BP use after the discontinuation of denosumab, but further high-quality randomized trials are required to prove this.

**Key points**

- Potent bisphosphonates reduced incident vertebral fracture risk by 71% overall.
- Potent bisphosphonates versus no subsequent therapy reduced vertebral fracture risk by 78%.
- Multiple vertebral fractures were reduced by 92% with potent bisphosphonate therapy.
- Clinical vertebral fractures showed an 82% risk reduction favoring potent bisphosphonates.
- Evidence comparing potent bisphosphonates with SERMs was limited and inconclusive.

## 1. Introduction

Osteoporosis is a widespread skeletal condition characterized by low bone mineral density (BMD) and microarchitectural degradation, and is a common skeletal illness that puts afflicted people at risk for fragility fractures, which have a substantial global morbidity, mortality, and healthcare burden. Among the available pharmacological agents for osteoporosis management, denosumab has emerged as a highly effective therapy, and receptor activator of nuclear factor kappa-B ligand (RANKL) is the target of this completely human monoclonal antibody. In clinical trials and real-world settings, it has shown sustained increases in BMD and clinically meaningful reductions in vertebral, non-vertebral, and hip fracture risk.^[1]^

However, stopping denosumab medication has created a major and well-known problem. In contrast to bisphosphonates (BPs), which irreversibly bind to hydroxyapatite in bone and exert antiresorptive effects for months to years after treatment has been stopped, the action of denosumab is fully reversible. RANKL-mediated osteoclast activity rebounds substantially 12 to 24 months after stopping medication and leads to rapid and marked loss of BMD, often exceeding the gains achieved during treatment. Most significantly, a number of clinical reports, observational studies, and registries have shown that withdrawing denosumab contributes to a higher risk of multiple vertebral fractures, a condition now known as “rebound-associated vertebral fractures.” This rebound effect is not only clinically significant but potentially life-changing, as multiple simultaneous vertebral fractures can lead to severe pain, deformity, loss of height, and significantly reduced quality of life.^[2]^ Clinical guidelines and expert consensus statements increasingly recommend the sequential use of antiresorptive drugs, especially potent BPs, as follow-on therapy after denosumab discontinuation to limit or prevent the rebound phenomenon. Of the BPs, the most clinically studied are zoledronate (ZOL), a potent intravenous BP with a once-yearly dose, and alendronate (ALN), an oral nitrogen-containing BP administered once weekly. Both drugs are reasonable pharmacological bridges after denosumab discontinuation, inhibiting farnesyl pyrophosphate synthase in osteoclasts, leading to cell death and reduced bone resorption in a RANKL-independent manner.^[3]^

This sequential approach is biologically valid and is increasingly accepted clinically, although the data supporting it are inconsistent. Differences in the BP drug used, the number of doses administered, the duration of follow-up, the timing of initiation relative to denosumab discontinuation, and the patient populations studied are notable. There are few comparative studies comparing ALN and ZOL in this specific context, and the best way forward has not yet been decided by a conclusive randomized controlled study. Moreover, the narrative reviews and expert commentary that are currently available have not used rigorous, systematic, and quantitative approaches to synthesize the available information or to formally assess its certainty, although they are useful.^[4,5]^

To close this evidence gap, the present study performed a systematic review and meta-analysis of the existing literature investigating the efficacy of potent BP therapy (ALN and ZOL) as sequential follow-on treatment after denosumab discontinuation in adults with osteoporosis or low bone mass. Comparators included no further therapy and non-BP alternatives, including selective estrogen receptor modulators (SERMs) such as raloxifene and bazedoxifene. Outcomes of interest included incident vertebral fracture, clinical vertebral fracture, multiple vertebral fractures, any fracture, and non-vertebral fracture after denosumab discontinuation.

## 2. Methods

The Preferred Reporting Items for Systematic Reviews and Meta-Analyses criteria were followed in the conduct of this systematic review and meta-analysis.^[6]^ Ethical approval and patient consent were not required because this systematic review and meta-analysis was based entirely on previously published studies.

### 2.1. Literature search and search strategy

A systematic literature search was performed from the development of the databases to May 2026 using PubMed (MEDLINE), Scopus, the Cochrane Library (CENTRAL), and ClinicalTrials.gov. The search strategy was constructed around 3 main concepts connected by the Boolean operator AND: discontinuation of denosumab (denosumab, Prolia, discontinuation, cessation, withdrawal, stop, and rebound), potent BP therapy (ALN, zoledronic acid, BP, potent BP, and sequential therapy), and fracture outcomes (fracture*, vertebral fracture, multiple vertebral fracture, rebound-associated vertebral fracture, and osteoporotic fracture). Controlled vocabulary (Medical Subject Headings terms in PubMed) and free-text keywords were used, the latter with appropriate syntax applied to the different databases. There were no limitations on language, publication date, or publication status. The complete database-specific search strategies are provided in Table S1, Supplemental Digital Content 1.

### 2.2. Inclusion and exclusion criteria

Studies eligible for inclusion comprised individuals aged ≥18 years with osteoporosis or low bone mass who discontinued denosumab and were treated with potent BP therapy, defined as zoledronic acid 5 mg intravenously or ALN 70 mg orally once weekly. Comparator groups included individuals who received no subsequent antiresorptive medication, calcium and vitamin D supplements, or a SERM like raloxifene or bazedoxifene. Studies comparing potent BPs with other BPs, such as risedronate or ibandronate, were excluded from the primary meta-analysis because they did not directly address the prespecified comparison. Studies with mixed drug classes were excluded unless extractable data were available separately for ALN or zoledronic acid arms. Studies had to provide at least 1 prespecified fracture outcome with extractable dichotomous data, described as the number of occurrences and the total number of participants in each treatment arm. Included were full-text publications from retrospective observational studies, prospective cohort studies, and randomized controlled trials. Excluded were case reports, case series with <10 patients per arm, editorials, narrative reviews, systematic reviews, conference abstracts without numerical outcomes, in vitro research, and animal experiments.

### 2.3. Study screening and data extraction

Before being screened, all obtained records were entered into a reference management system, and duplicate entries were eliminated. Two independent reviewers screened the titles and abstracts of all identified records to exclude clearly irrelevant studies. Full texts of potentially eligible articles were then retrieved and assessed against the prespecified inclusion and exclusion criteria. Discussions or consultations with a third reviewer were used to settle any disputes among reviewers.

A standardized form created prior to the review was used to extract data. The following variables were obtained: study characteristics (first author, year, country, study design, and sample size); patient demographics and clinical features (age, sex, baseline BMD, and fracture history); intervention details (BP agent, dose, route, and duration); comparator details; and reported outcomes. The primary outcome was incident vertebral fracture after denosumab withdrawal. Among the secondary outcomes were clinical vertebral fractures, multiple vertebral fractures, any fracture, and non-vertebral fractures. The clinically reported fractures were aggregated together to create the any-fracture category; asymptomatic vertebral fracture assessment fractures only, on the other hand, were maintained within the incident vertebral fractures category without being double-counted in the any-fracture category. The number of occurrences and total participants in each treatment arm were extracted as outcome data. Disagreements were settled by consensus after the 2 reviewers separately extracted the data.

### 2.4. Risk of bias assessment

The Newcastle–Ottawa Scale (NOS), a tool developed specifically for observational research, was used to evaluate the methodological quality of all included studies,^[7]^ while the randomized trial was assessed using revised Cochrane risk-of-bias tool for randomized trials (RoB 2; Cochrane, London, UK). The NOS assesses studies in 3 areas: study group selection, cohort comparability, and ascertainment of exposure and outcome. Based on its overall score, each study was categorized as having a low, moderate, or high risk of bias. Disagreements were settled by discussion or consultation with a third reviewer after 2 reviewers independently finished the assessment. Another tool utilized to measure the quality of the evidence for each outcome is called the Grading of Recommendations, Assessment, Development, and Evaluation (GRADE), which evaluates the overall body of evidence as high, moderate, low, or very low quality.

### 2.5. Outcomes

The primary efficacy outcome was incident vertebral fracture after denosumab discontinuation. Secondary outcomes included clinical vertebral fracture, multiple vertebral fractures, any fracture, and non-vertebral fracture. Clinical vertebral fracture was defined as a symptomatic vertebral fracture confirmed radiologically or by imaging in the setting of back pain or functional impairment, according to the definitions used in the original publications. For the any-fracture outcome, clinically reported fracture events were pooled; asymptomatic vertebral fracture assessment-only vertebral fractures were retained under the incident vertebral fracture outcome and were not double-counted in the any-fracture analysis.

Secondary outcomes included multiple vertebral fractures (2 or more vertebral fractures in the same patient during the follow-up period) and non-vertebral fractures (fractures occurring at skeletal sites other than the vertebrae, including hip, wrist, and other peripheral sites).

Outcome data were extracted as the number of events and total participants per treatment arm (potent BP vs comparator) for each fracture category. Outcome definitions from the original publications were retained; therefore, clinical and measurement heterogeneity were expected and addressed through subgroup analyses and narrative interpretation.

### 2.6. Statistical analysis

Risk ratios (RRs) with 95% confidence intervals (CIs) were pooled after fracture outcomes were evaluated as dichotomous data. For statistical analysis, a Mantel–Haenszel random-effects model was employed to account for methodological and clinical diversity among trials.^[8,9]^ Cochran’s χ^2^ test and the *I*^2^ statistic were used to analyze heterogeneity; low, moderate, and high heterogeneity were defined as 25%, 50%, and 75%, respectively.^[10]^ For every outcome based on the comparator group, 2 prespecified subgroup analyses were carried out: potent BP versus SERM and potent BP versus no further medication. A subgroup difference test was run. Review Manager (RevMan; The Cochrane Collaboration, London, UK) software was used for all two-tailed analyses, with *P* < .05 considered statistically significant.

### 2.7. Certainty of evidence assessment

Two separate writers evaluated the certainty of the evidence in this meta-analysis using the GRADE technique, classifying it from high to very low.^[11,12]^ A third author discussed and reached a consensus to settle any disputes.

## 3. Results

### 3.1. Study selection

Database searches identified 726 records: PubMed (n = 400), Scopus (n = 246), Cochrane Library (n = 69), and ClinicalTrials.gov (n = 11). Once 250 of them were removed as duplicates, there were 476 remaining for screening of titles and abstracts.

Of these, 423 documents were excluded during the screening stage because they did not meet the inclusion criteria and were not eligible for full-text retrieval; 53 documents were requested for full-text retrieval. Retrieval and assessment of all 53 reports were successful.

After full-text review, 47 reports were excluded because there was no clear post-denosumab BP transition (n = 23), no fracture outcome data reported (n = 15), and the wrong comparator was used: continuation of treatment versus no BP bridging (n = 9).

Finally, 6 studies were included in the systematic review. The study-selection process is presented in the OA Guidelines flow diagram.

### 3.2. Characteristics of included studies

This meta-analysis included 6 studies published between 2021 and 2025, comprising 1 open-label randomized controlled trial (Ramchand et al^[13]^) and 5 retrospective observational studies (Tutaworn et al,^[14]^ Ha et al,^[15]^ Grassi et al,^[16]^ Everts-Graber et al,^[17]^ and Ebina et al^[18]^). The studies were conducted in 5 countries: the United States (n = 2), South Korea (n = 1), Italy (n = 1), Switzerland (n = 1), and Japan (n = 1). Both single-center and multicenter research designs were included in the review. In all studies, patients diagnosed with osteoporosis were treated with denosumab and then subsequently withdrew from it. The intervention arms used for the primary quantitative synthesis were limited to potent BP therapy, defined as zoledronic acid 5 mg intravenously or ALN 70 mg/week orally. Risedronate, ibandronate, low-dose ALN/calcitriol, and mixed oral BP arms were considered among the characteristics of each study but excluded from quantitative synthesis in the primary analysis unless the results related to ALN or zoledronic acid could be extracted. Furthermore, 2 studies evaluated a comparison with SERM therapies, including raloxifene or bazedoxifene. The number of patients in individual arms varied from 11 to 171; the number of patients in no-treatment comparator arms varied from 19 to 33. Vertebral fractures at baseline showed significant variability among arms, ranging between 0% and 15.8%. Vertebral fracture incidence following discontinuation of denosumab treatment was used as the primary outcome variable in all included studies. Secondary outcomes included multiple vertebral fractures, clinical vertebral fractures, non-vertebral fractures, and all fractures. Detailed study and participant characteristics are presented in Tables 1 and 2.

**Table 1:** Study characteristics.

Study ID	Country	Study type	Intervention	Control	Sample size (intervention)	Sample size (control)	Male (%) intervention	Male (%) control
Tutaworn et al^[14]^	USA	Retrospective cohort	Risedronate (RIS) 35 mg/wk	No treatment (NT)	22	33	0	3.00
			Alendronate (ALN) 70 mg/wk	No treatment (NT)	34	33	11.80	3.00
			Zoledronic acid (ZOL) 5 mg single dose	No treatment (NT)	32	33	12.50	3.00
Ramchand et al^[13]^	USA	RCT (open-label)	Alendronate 70 mg/wk (after denosumab)	Raloxifene 60 mg/d (after denosumab)	26	25	0	0
Ha et al^[15]^	Korea	Retrospective multicenter observational	Low-dose alendronate/calcitriol (MXM)	N/A (multiple groups compared)	118	–	6.80	–
			Alendronate (ALN) full-dose	N/A (multiple groups compared)	53	–	5.70	–
			Risedronate (RIS)	N/A (multiple groups compared)	20	–	5.00	–
			Ibandronate (IBN)	N/A (multiple groups compared)	30	–	0	–
			Zoledronic acid (ZOL)	N/A (multiple groups compared)	106	–	5.70	–
			SERM (raloxifene/bazedoxifene)	N/A (multiple groups compared)	33	–	0	–
Grassi et al^[16]^	Italy	Retrospective monocentric	Bisphosphonate (alendronate or zoledronate)	No treatment	101	19	5.90	15.80
			Alendronate (ALN)	Zoledronic acid (ZOL)	28	73	Not reported	Not reported
Everts-Graber et al^[17]^	Switzerland	Retrospective observational	Zoledronate (ZOL) single infusion	No subsequent treatment	171	26	0	0
			Other bisphosphonates/SERM (alendronate, ibandronate, SERM)	No subsequent treatment	22	26	0	0
Ebina et al^[18]^	Japan	Retrospective multicenter	Raloxifene (RAL)	N/A (compared to BP groups)	13	–	0	–
			Weekly/monthly bisphosphonate (wmBP: ALN, RIS, IBN)	N/A (compared to RAL and ZOL)	29	–	0	–
			Zoledronic acid (ZOL)	N/A (compared to RAL and wmBP)	11	–	0	–

BP = bisphosphonate, SERM = selective estrogen receptor modulator.

**Table 2:** Patient’s characteristics.

Study ID	Group	Age (yr)	Female (%)	BMI (kg/m^2^)	Prior vertebral Fx (%)	Prior any Fx (%)	Prior BP use (%)	Dmab injections (mean)	LS T-score (or BMD)	FN T-score (or BMD)	TH T-score (or BMD)
Tutaworn et al^[14]^	No treatment (NT)	72.2 ± 9.0	97	22.3 ± 2.8	15.2	63.6	36.4	4.6 ± 1.6	−1.9 ± 1.1	−2.2 ± 0.7	−1.9 ± 0.6
	Risedronate (RIS)	68.7 ± 8.2	100	22.7 ± 2.9	9.1	50	50	5.2 ± 2.4	−1.7 ± 0.6	−1.8 ± 0.5	−1.5 ± 0.4
	Alendronate (ALN)	72.0 ± 7.5	88.2	24.3 ± 4.9	14.7	61.8	44.1	6.5 ± 2.8	−1.3 ± 1.0	−1.8 ± 0.7	−1.5 ± 0.6
	Zoledronic acid (ZOL)	71.0 ± 7.5	87.5	23.2 ± 3.0	12.5	68.8	25	5.3 ± 2.6	−1.3 ± 0.7	−1.8 ± 0.5	−1.5 ± 0.7
Ramchand et al^[13]^	Dmab → Alendronate	66.0 ± 5.1	100	23.2 ± 3.3	–	38.0^1^	38	2 (fixed)	0.794 ± 0.085 g/cm^2^†	0.585 ± 0.050 g/cm^2^†	0.707 ± 0.068 g/cm^2^†
	Dmab → Raloxifene	65.4 ± 5.3	100	24.6 ± 3.1	–	40.0^1^	28	2 (fixed)	0.804 ± 0.072 g/cm^2^†	0.628 ± 0.071 g/cm^2^†	0.736 ± 0.072 g/cm^2^†
Ha et al^[15]^	Low-dose ALN/calcitriol (MXM)	66.4 ± 8.7	93.2	23.5 ± 3.0	–	16.9	–	4.6 ± 2.4	−1.8 ± 0.8	−1.7 ± 0.7	−1.4 ± 0.8
	Alendronate (ALN)	65.8 ± 9.4	94.3	23.3 ± 3.2	–	34	–	4.1 ± 1.9	−1.9 ± 0.8	−1.7 ± 0.7	−1.5 ± 0.8
	Risedronate (RIS)	68.7 ± 8.8	95	24.2 ± 4.9	–	35	–	3.9 ± 1.8	−1.8 ± 1.0	−1.9 ± 0.6	−1.5 ± 0.6
	Ibandronate (IBN)	66.8 ± 7.6	100	24.0 ± 3.9	–	13.3	–	4.3 ± 2.0	−1.9 ± 0.7	−1.7 ± 0.8	−1.4 ± 0.8
	Zoledronic acid (ZOL)	69.0 ± 9.3	94.3	23.8 ± 3.6	–	37.7	–	4.6 ± 2.0	−2.0 ± 1.0	−2.1 ± 0.8	−1.7 ± 0.8
	SERM (raloxifene/bazedoxifene)	67.8 ± 6.8	100	22.0 ± 3.1	–	9.1	–	3.9 ± 1.9	−2.0 ± 0.5	−1.9 ± 0.6	−1.6 ± 0.8
Grassi et al^[16]^	Treated (alendronate/ZOL)	70.0 ± 10.6	94.1	24.0 ± 3.9	85.1	87.1	70.3	6.6 ± 2.6	−2.4 ± 1.1	−2.3 ± 0.8	−2.0 ± 0.8
	Non-treated control	63.5 ± 15.0	84.2	24.5 ± 3.9	68.4	73.7	42.1	3.6 ± 1.8	−1.9 ± 1.7	−2.2 ± 0.7	−1.8 ± 0.9
Everts-Graber et al^[17]^	No subsequent treatment	65 ± 7.9	100	24 ± 4.1	31	–	31	5.0 (5.0–7.0)‡	–	–	–
	Zoledronate (ZOL)	66 ± 7.8	100	24 ± 3.8	30	–	41	5.0 (5.0–7.0)‡	–	–	–
	Other BPs/SERM	67 ± 8.0	100	23 ± 3.7	45	–	32	5.5 (5.0–8.0)‡	–	–	–
Ebina et al^[18]^	Raloxifene (RAL)	73.1 (overall mean)	100	20.5 (overall)	50.9 (overall)	–	–	2.5 ± 1.1	−2.7 (overall)	−2.2 (overall)	–
	Weekly/monthly BP (wmBP)	73.1 (overall mean)	100	20.5 (overall)	50.9 (overall)	–	–	2.4 ± 1.5	−2.7 (overall)	−2.2 (overall)	–
	Zoledronic acid (ZOL)	73.1 (overall mean)	100	20.5 (overall)	50.9 (overall)	–	–	3.3 ± 2.3	−2.7 (overall)	−2.2 (overall)	–

The superscript ^1^, †, and ‡ indicate specific data characteristics and reporting formats.

BMD = bone mineral density, BMI = body mass index, BP = bisphosphonate, Dmab = denosumab, FN = femoral neck, Fx = fracture, LS = lumbar spine, SERM = selective estrogen receptor modulator, TH = total hip.

### 3.3. Risk of bias and methodological quality assessment

NOS was used to assess the methodological quality of the included observational studies. Five of the 6 included studies were observational. Among these, Everts-Graber et al^[17]^ and Grassi et al^[16]^ were rated as high quality with NOS scores of 9, while Ha et al^[15]^ and Tutaworn et al^[14]^ were rated as high quality with NOS scores of 8. Ebina et al^[18]^ was rated as moderate quality with a score of 7 because of limitations in comparability and outcome assessment. None of the observational studies were judged to be low quality. NOS assessments are summarized in Table 3.

**Table 3:** Newcastle–Ottawa Scale (NOS) scores for included studies.

Study	Newcastle–Ottawa Scale				
	Selection	Comparability	Outcome	Total score	Study quality
Ebina et al^[18]^	4	1	2	7	Moderate
Everts-Graber et al^[17]^	4	2	3	9	High
Grassi et al^[16]^	4	2	3	9	High
Ha et al^[15]^	3	2	3	8	High
Tutaworn et al^[14]^	4	2	2	8	High

**Figure 1.**
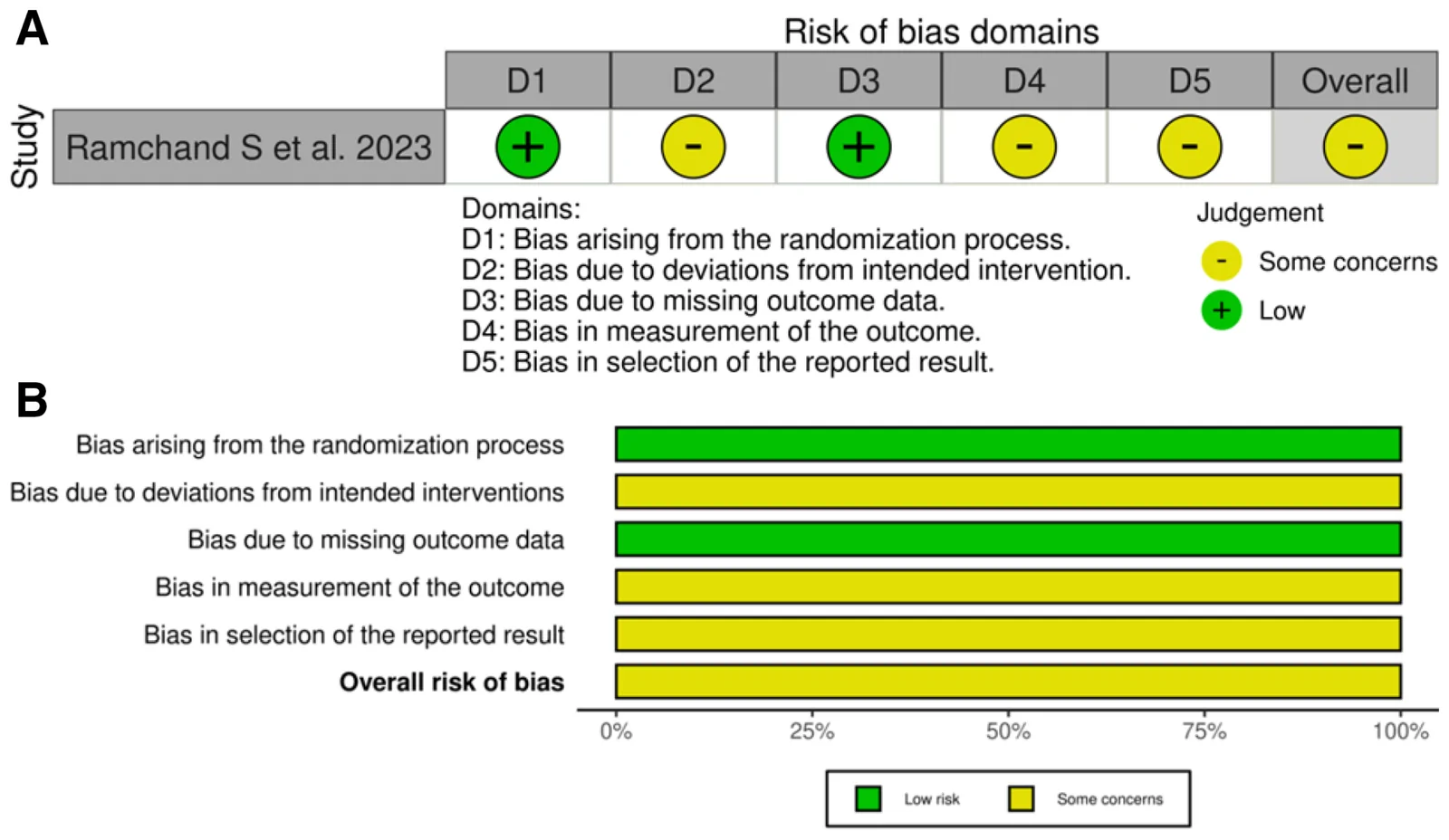
Risk-of-bias assessment of the included randomized controlled trial. (A) Traffic-light plot showing domain-level risk-of-bias judgments for the included randomized controlled trial using the revised Cochrane risk-of-bias tool for randomized trials. Green indicates low risk, yellow indicates some concerns, and red indicates high risk. (B) Weighted bar chart summarizing the proportion of RoB 2 judgments classified as low risk, some concerns, or high risk across domains. RoB 2 = revised Cochrane risk-of-bias tool for randomized trials.

The ROB 2 tool was used to evaluate the comparative study by Ramchand et al^[13]^ and the results showed an overall rating of “some concerns,” indicating moderate methodological quality. The randomization procedure and the management of missing outcome data in the study demonstrated a low risk of bias. However, because of its open-label nature, there were several issues with outcome measurement and variations from the planned interventions. Additionally, concerns were noted in the selection of reported results. Importantly, the study was not judged to be at high risk of bias in any domain. The detailed RoB 2 assessment is presented in Figure 1A and B.

### 3.4. Certainty of evidence

The quality of the evidence for a variety of important outcomes was evaluated using the GRADE method. Low to very low certainty was assigned to the evidence associated with incident vertebral fractures, multiple vertebral fractures, clinical vertebral fractures, any fractures, and non-vertebral fractures. This classification predominantly arose from the observational nature of the studies analyzed. The primary reasons for downgrading the evidence included imprecision, particularly in outcomes characterized by broad CIs or a limited number of studies. Additionally, no noticeable issues with publication bias or indirectness were found for any of the evaluated outcomes. The complete GRADE assessment is provided in Table S2, Supplemental Digital Content 2.

### 3.5. Quantitative synthesis (meta-analysis results)

#### 3.5.1. Incidence of vertebral fracture

##### 3.5.1.1. Overall effect

**Figure 2.**
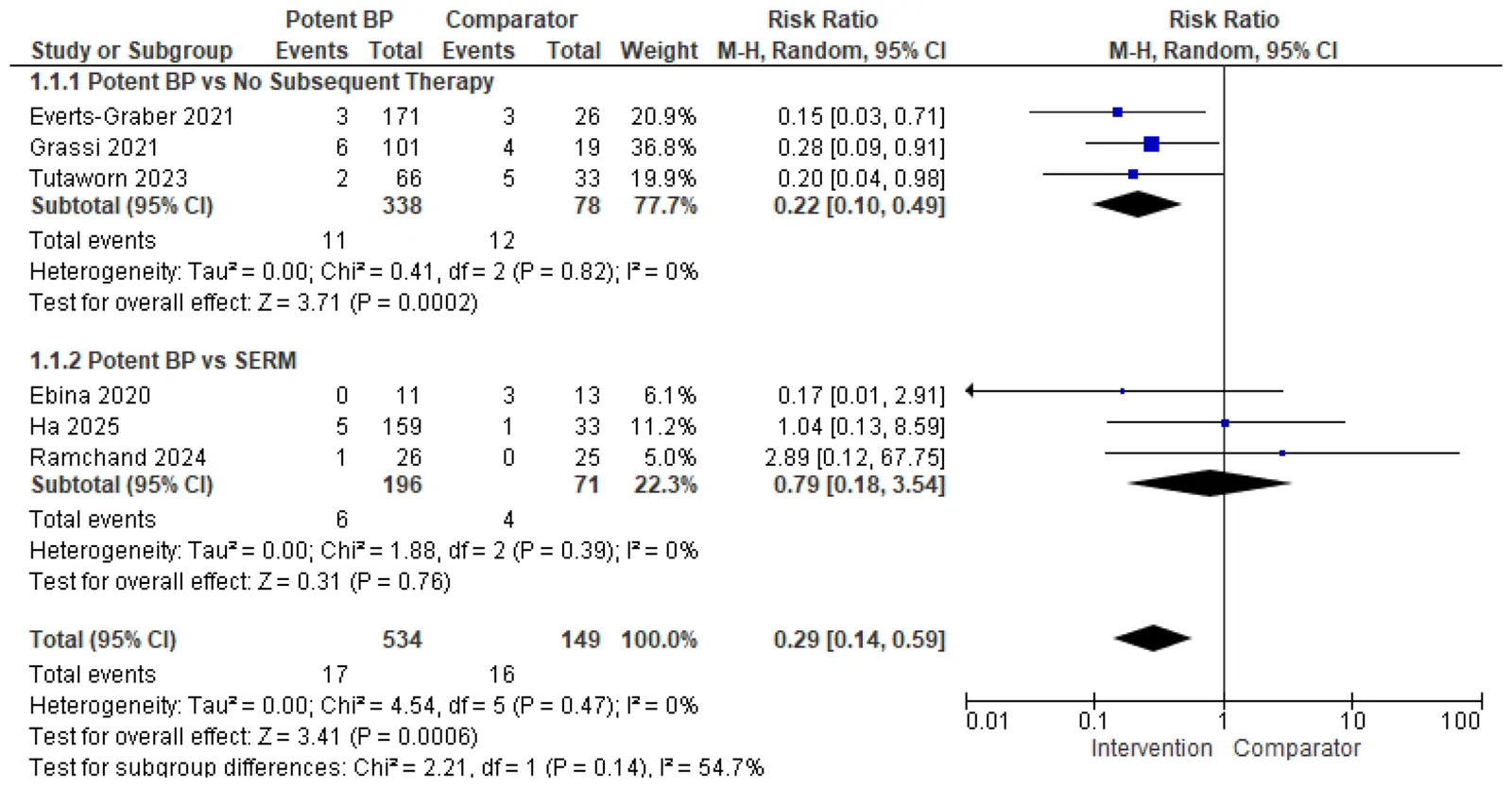
Forest plot of incident vertebral fracture after denosumab discontinuation, stratified by comparator type. BP = bisphosphonate, CI = confidence interval, M-H = Mantel–Haenszel, SERM = selective estrogen receptor modulator.

Six studies were included in the Mantel–Haenszel random-effects meta-analysis, including 534 people in the potent BP group and 149 participants in the comparison group. When compared with non-BP comparators, potent BP medication was linked to a statistically significant decrease in incident vertebral fractures (RR = 0.29; 95% CI: 0.14–0.59; *Z* = 3.43; *P* = .0006), or a 71% relative risk reduction. Overall heterogeneity was absent (*I*^2^ = 0%; Tau^2^ = 0.00; Chi^2^ = 4.54, df = 5, *P* = .47; Fig. 2).

##### 3.5.1.2. Subgroup 1.1.1: potent BP versus subsequent no therapy

Three studies compared potent BP therapy with no subsequent therapy, enrolling 338 participants in the potent BP group and 78 participants in the no-therapy comparator group. The pooled estimate showed a statistically significant reduction in vertebral fracture risk favoring potent BP therapy (RR = 0.22; 95% CI: 0.10–0.49; *Z* = 3.71; *P* = .0002), corresponding to a 78% relative risk reduction. Heterogeneity within this subgroup was absent (*I*^2^ = 0%; Tau^2^ = 0.00; Chi^2^ = 0.41, df = 2, *P* = .82).

##### 3.5.1.3. Subgroup 1.1.2: potent BP versus SERM

Potent BP therapy and SERM therapy were compared in 3 studies, including 196 people in the potent BP group and 71 in the SERM comparator group. No statistically significant difference was observed between groups (RR = 0.79; 95% CI: 0.18–3.54; *Z* = 0.31; *P* = .76). Heterogeneity within this subgroup was low (*I*^2^ = 5%; Tau^2^ = 0.09; Chi^2^ = 2.10, df = 2, *P* = .35).

##### 3.5.1.4. Test for subgroup differences

The test for subgroup differences did not reach statistical significance (Chi^2^ = 2.21, df = 1, *P* = .14; *I*^2^ = 54.7%), although the point estimates suggested a stronger protective effect when potent BPs were compared with no subsequent therapy than when compared with SERM therapy.

#### 3.5.2. Multiple vertebral fractures

##### 3.5.2.1. Overall effect

**Figure 3.**
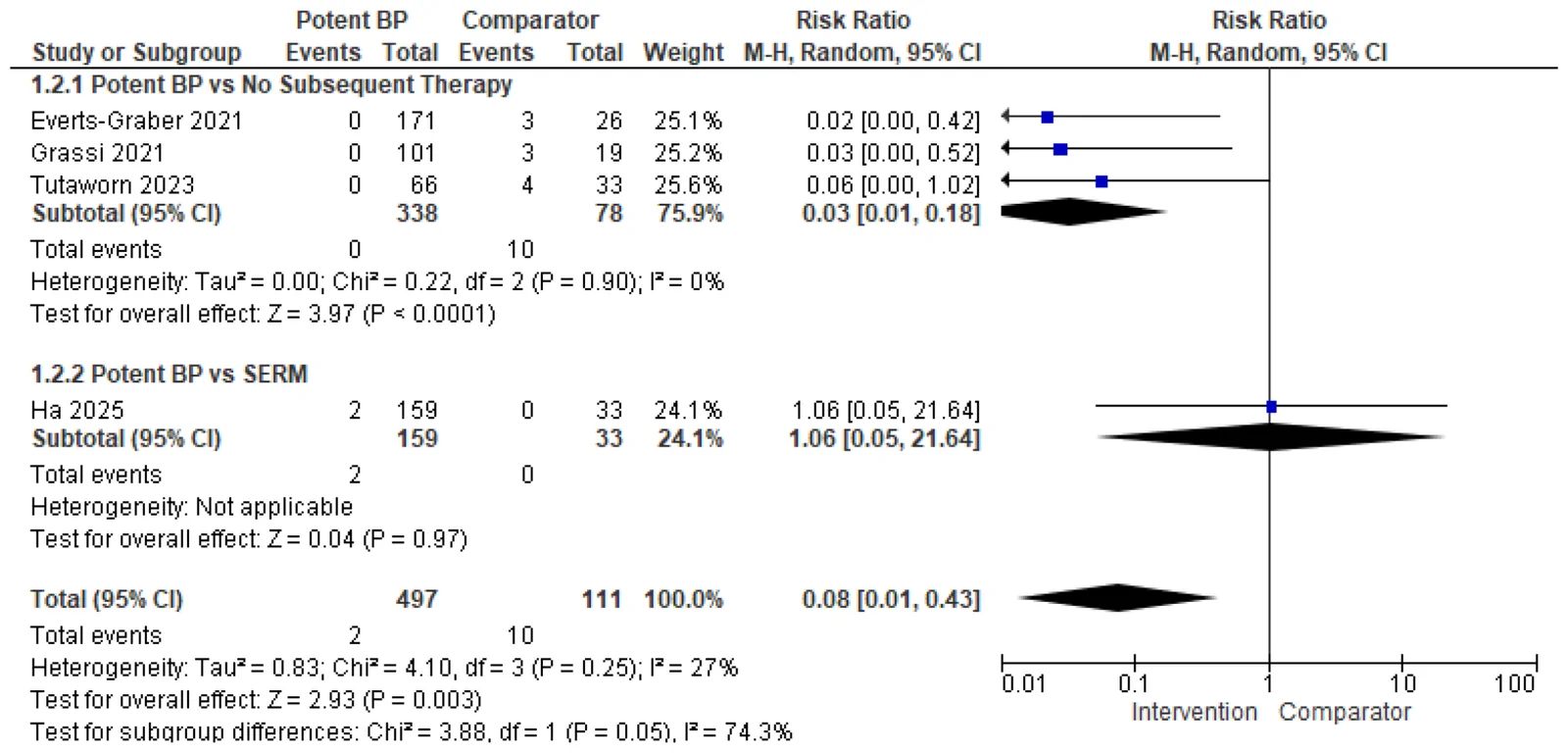
Forest plot of multiple vertebral fractures after denosumab discontinuation, stratified by comparator type. BP = bisphosphonate, CI = confidence interval, M-H = Mantel–Haenszel, SERM = selective estrogen receptor modulator.

Four studies (n = 497 intervention; n = 111 comparator) were included in the meta-analysis for multiple vertebral fractures, using a random-effects model (Mantel–Haenszel). Potent BP therapy was associated with a significant 92% reduction in the risk of multiple vertebral fractures compared with all comparators combined (RR = 0.08; 95% CI: 0.01–0.43; *Z* = 2.93; *P* = .003). Between-study heterogeneity was moderate (*I*^2^ = 27%; Tau^2^ = 0.83; Chi^2^ = 4.10, df = 3, *P* = .25; Fig. 3).

##### 3.5.2.2. Subgroup 1.2.1: potent BP versus no subsequent therapy

Three studies (Everts-Graber et al,^[17]^ Grassi et al,^[16]^ and Tutaworn et al^[14]^) contributed to this subgroup, with 338 participants receiving potent BP and 78 receiving no therapy. The pooled RR was 0.03 (95% CI: 0.01–0.18; *Z* = 3.97; *P* < .0001), representing a 97% relative risk reduction. Heterogeneity was absent (*I*^2^ = 0%; Tau^2^ = 0.00; Chi^2^ = 0.22, df = 2, *P* = .90).

##### 3.5.2.3. Subgroup 1.2.2: potent BP versus SERM

Ha et al^[15]^ contributed 159 participants in the potent BP group and 33 participants in the SERM comparator group. No significant difference was observed between potent BP and SERM in preventing multiple vertebral fractures (RR = 1.06; 95% CI: 0.05–21.64; *Z* = 0.04; *P* = .97). Heterogeneity was not applicable given the inclusion of only 1 study.

##### 3.5.2.4. Test for subgroup differences

The test for subgroup differences was statistically significant (Chi^2^ = 3.88, df = 1, *P* = .05), with very high between-subgroup variability (*I*^2^ = 74.3%).

#### 3.5.3. Clinical vertebral fractures

##### 3.5.3.1. Overall effect

**Figure 4.**
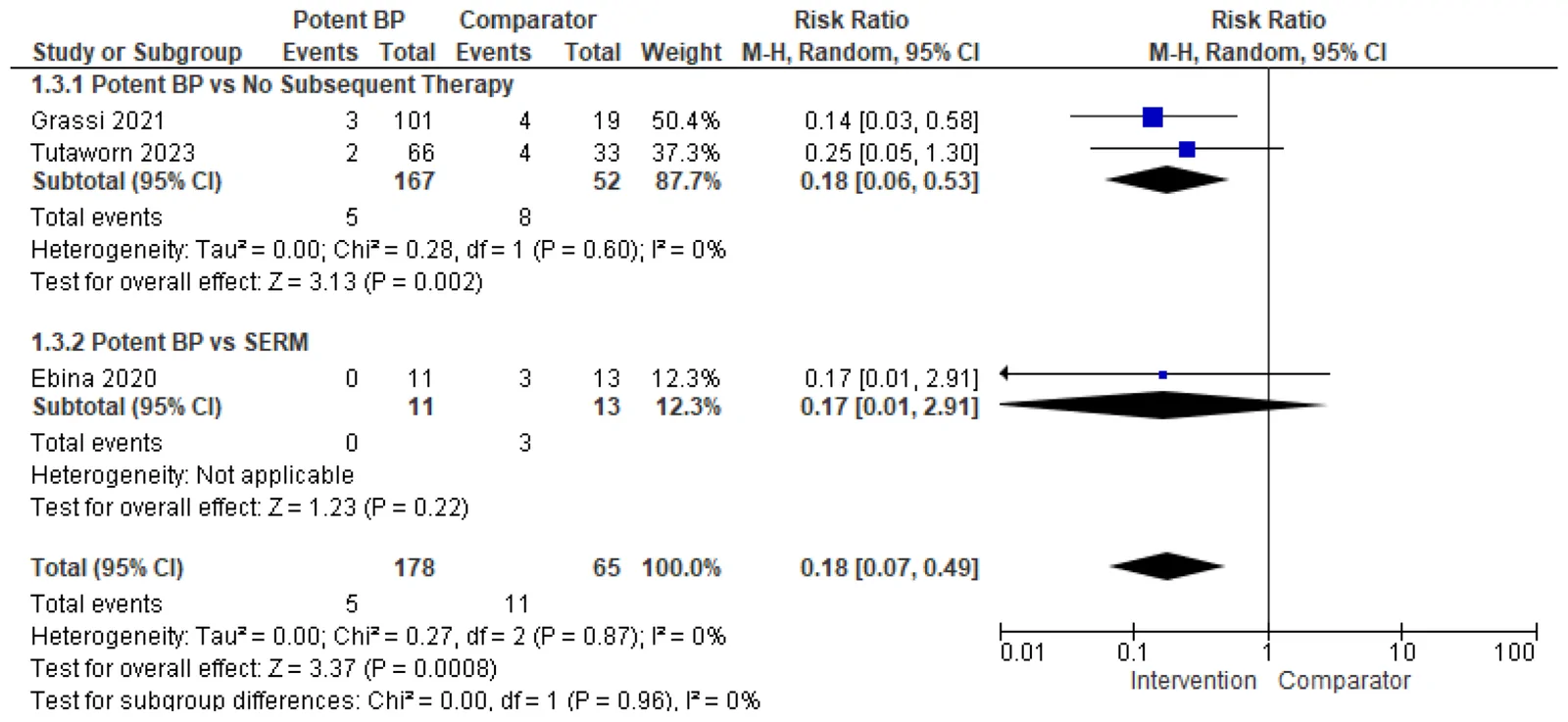
Forest plot of clinical vertebral fracture after denosumab discontinuation, stratified by comparator type. BP = bisphosphonate, CI = confidence interval, M-H = Mantel–Haenszel, SERM = selective estrogen receptor modulator.

Three studies (n = 178 intervention; n = 65 comparator) were pooled using a random-effects model (Mantel–Haenszel) to assess the effect of potent BP therapy on clinical vertebral fractures. The overall analysis demonstrated a statistically significant 82% reduction in risk favoring potent BP (RR = 0.18; 95% CI: 0.07–0.49; *Z* = 3.37; *P* = .0008). Between-study heterogeneity was negligible (*I*^2^ = 0%; Tau^2^ = 0.00; Chi^2^ = 0.27, df = 2, *P* = .87; Fig. 4).

##### 3.5.3.2. Subgroup 1.3.1: potent BP versus no subsequent therapy

Two studies (Grassi et al^[16]^ and Tutaworn et al^[14]^) contributed to this subgroup, enrolling 167 participants in the potent BP arm and 52 in the no-therapy arm. A significant 82% relative risk reduction was observed (RR = 0.18; 95% CI: 0.06–0.53; *Z* = 3.13; *P* = .002). Heterogeneity was absent (*I*^2^ = 0%; Tau^2^ = 0.00; Chi^2^ = 0.28, df = 1, *P* = .60).

##### 3.5.3.3. Subgroup 1.3.2: potent BP versus SERM

A single study (Ebina et al^[18]^; n = 11 intervention; n = 13 comparator) contributed to this subgroup. The point estimate favored potent BP; the result did not reach statistical significance (RR = 0.17; 95% CI: 0.01–2.91; *Z* = 1.23; *P* = .22). Heterogeneity assessment was not applicable given the inclusion of only 1 study.

##### 3.5.3.4. Test for subgroup differences

No statistically significant subgroup interaction was detected between the 2 comparator categories (Chi^2^ = 0.00, df = 1, *P* = .96; *I*^2^ = 0%).

#### 3.5.4. Any fracture

##### 3.5.4.1. Overall effect

**Figure 5.**
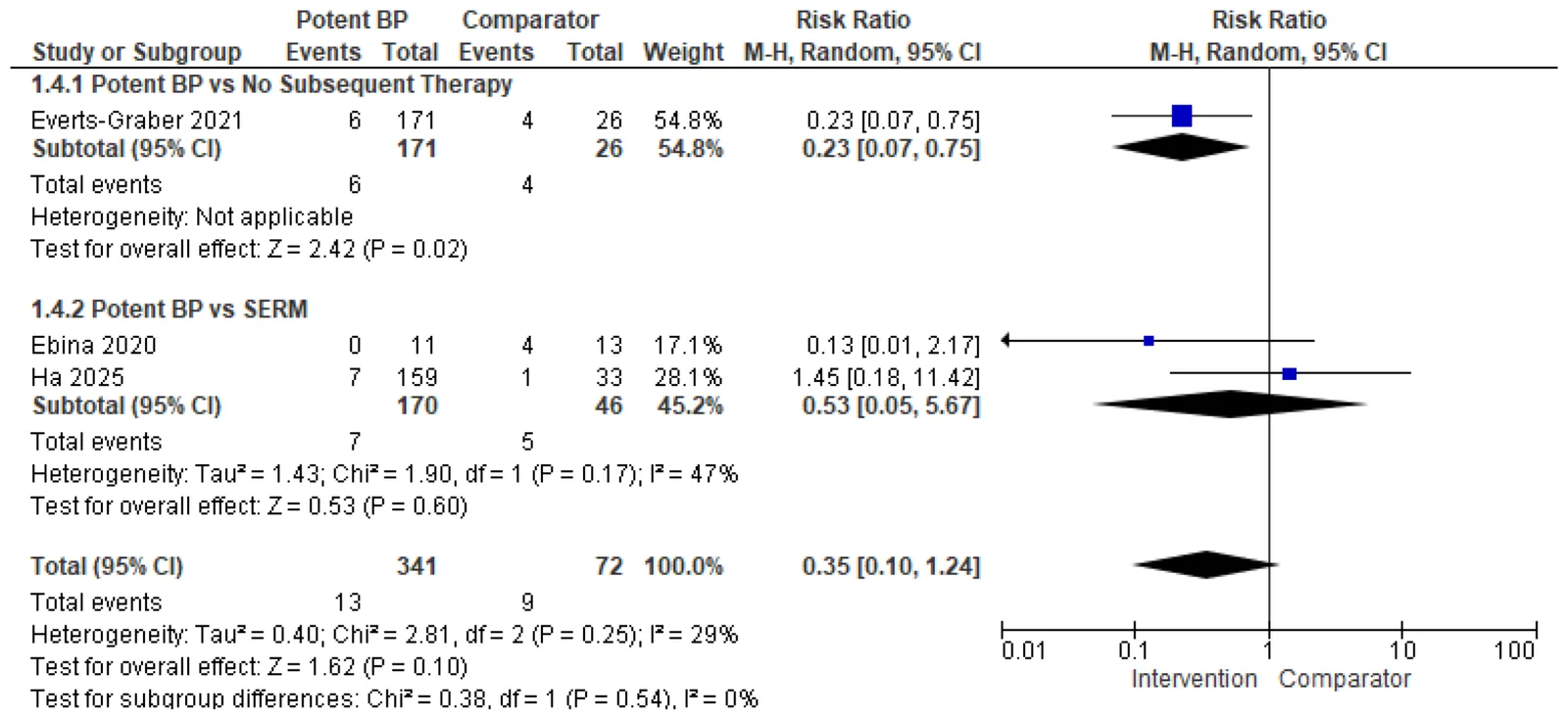
Forest plot of any fracture after denosumab discontinuation, stratified by comparator type. BP = bisphosphonate, CI = confidence interval, M-H = Mantel–Haenszel, SERM = selective estrogen receptor modulator.

Three studies (n = 341 intervention; n = 72 comparator) were included in a random-effects meta-analysis (Mantel–Haenszel) for the composite outcome of any fracture. The pooled RR did not reach statistical significance (RR = 0.35; 95% CI: 0.10–1.24; *Z* = 1.62; *P* = .10), though the point estimate consistently favored potent BP therapy. Moderate between-study heterogeneity was observed (*I*^2^ = 29%; Tau^2^ = 0.40; Chi^2^ = 2.81, df = 2, *P* = .25; Fig. 5).

##### 3.5.4.2. Subgroup 1.4.1: potent BP versus no subsequent therapy

A single study (Everts-Graber et al^[17]^; n = 171 intervention; n = 26 comparator) contributed to this subgroup. A statistically significant 77% reduction in any fracture risk was demonstrated with potent BP (RR = 0.23; 95% CI: 0.07–0.75; *Z* = 2.42; *P* = .02). Heterogeneity was not applicable given the single-study contribution.

##### 3.5.4.3. Subgroup 1.4.2: potent BP versus SERM

Two studies (Ebina et al^[18]^ and Ha et al^[15]^) contributed to this subgroup, enrolling 170 participants in the potent BP arm and 46 in the SERM arm. The pooled estimate did not reach statistical significance (RR = 0.53; 95% CI: 0.05–5.67; *Z* = 0.53; *P* = .60). Substantial heterogeneity was present within this subgroup (*I*^2^ = 47%; Tau^2^ = 1.43; Chi^2^ = 1.90, df = 1, *P* = .17).

##### 3.5.4.4. Test for subgroup differences

No significant interaction was detected between the no-therapy and SERM subgroups (Chi^2^ = 0.38, df = 1, *P* = .54; *I*^2^ = 0%).

#### 3.5.5. Non-vertebral fractures

##### 3.5.5.1. Overall effect

**Figure 6.**
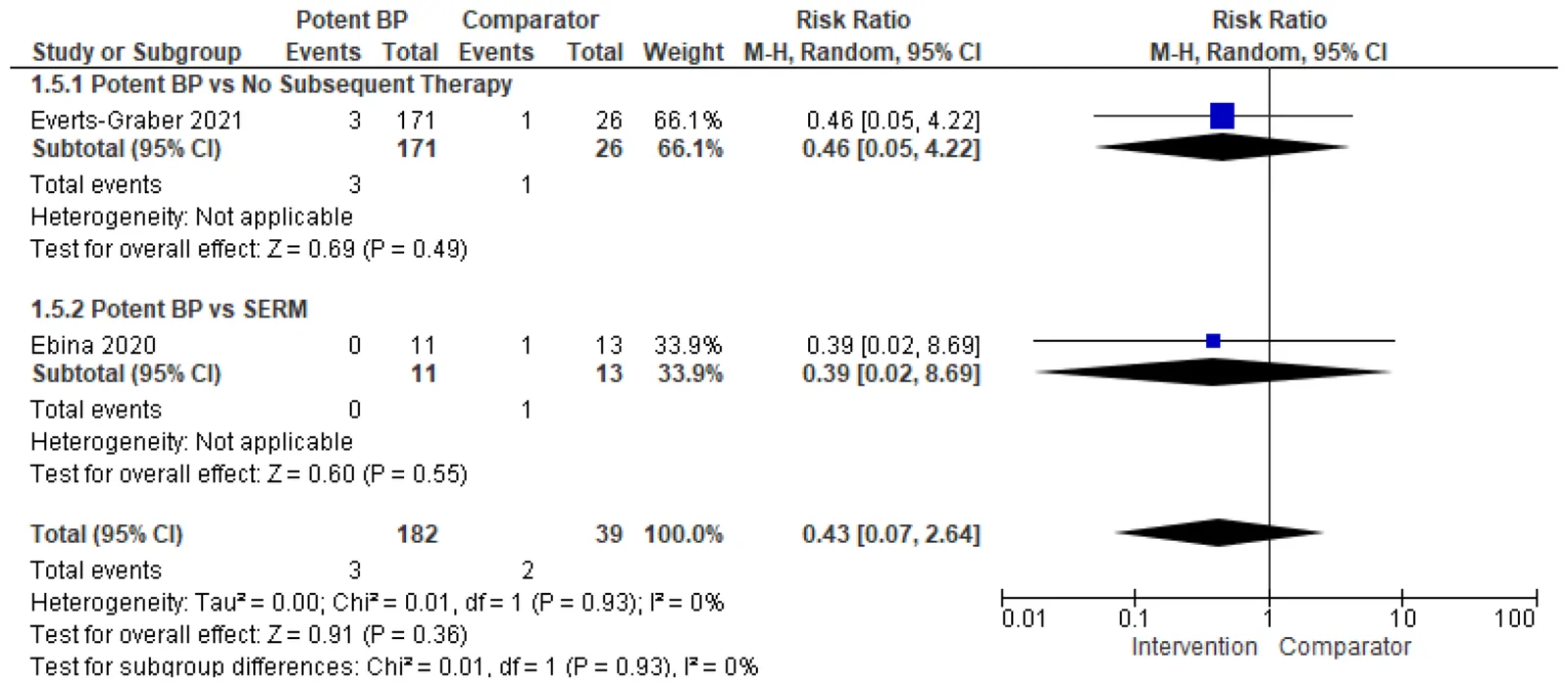
Forest plot of non-vertebral fracture after denosumab discontinuation, stratified by comparator type. BP = bisphosphonate, CI = confidence interval, M-H = Mantel–Haenszel, SERM = selective estrogen receptor modulator.

Two studies (n = 182 intervention; n = 39 comparator) were pooled using a random-effects model (Mantel–Haenszel) to evaluate the effect of potent BP therapy on non-vertebral fractures. The overall pooled RR did not reach statistical significance (RR = 0.43; 95% CI: 0.07–2.64; *Z* = 0.91; *P* = .36), though the point estimate directionally favored potent BP. Between-study heterogeneity was negligible (*I*^2^ = 0%; Tau^2^ = 0.00; Chi^2^ = 0.01, df = 1, *P* = .93; Fig. 6).

##### 3.5.5.2. Subgroup 1.5.1: potent BP versus no subsequent therapy

A single study (Everts-Graber et al^[17]^; n = 171 intervention; n = 26 comparator) contributed to this subgroup, accounting for 66.1% of the total weight. The pooled RR was 0.46 (95% CI: 0.05–4.22; *Z* = 0.69; *P* = .49), which did not achieve statistical significance. Heterogeneity was not applicable given the single-study contribution.

##### 3.5.5.3. Subgroup 1.5.2: potent BP versus SERM

A single study (Ebina et al^[18]^; n = 11 intervention; n = 13 comparator) contributed to this subgroup, representing 33.9% of the total analytical weight. The point estimate again favored potent BP, though without statistical significance (RR = 0.39; 95% CI: 0.02–8.69; *Z* = 0.60; *P* = .55). Heterogeneity was not applicable.

##### 3.5.5.4. Test for subgroup differences

No statistically significant interaction was identified between the no-therapy and SERM comparator subgroups (Chi^2^ = 0.01, df = 1, *P* = .93; *I*^2^ = 0%).

## 4. Discussion

This meta-analysis suggests that potent BP therapy after denosumab discontinuation is associated with a lower risk of incident vertebral fractures, multiple vertebral fractures, and clinical vertebral fractures compared with non-BP comparator strategies. There was no evident statistically significant change for any fracture or non-vertebral fracture. In contrast to comparisons with SERM therapy, which were constrained by small sample sizes, sparse occurrences, and large CIs, the protective effect seemed most noticeable when potent BPs were compared with no subsequent therapy. Overall, these findings support the clinical importance of sequential antiresorptive therapy after denosumab discontinuation, while also highlighting the need for higher-quality prospective evidence.

Denosumab is a strong antiresorptive drug, widely utilized in the management of osteoporosis due to its potent inhibition of osteoclast-mediated bone resorption and substantial improvement in BMD.^[19–21]^ Denosumab, in contrast to BPs, does not aggregate in the bone matrix, and skeletal benefits are quickly reversed upon withdrawal from the medication. This includes a rebound increase in bone turnover markers, accelerated bone loss, and an increased risk of vertebral fractures, especially multiple vertebral fractures.^[22,23]^ This “rebound phenomenon” has become a significant clinical concern over the last decade, and attention has grown into discovering the best sequential therapy after denosumab withdrawal.^[22]^ The long skeletal retention and extended antiresorptive activity of potent BPs, particularly zoledronic acid and ALN, have been suggested as a strategy to decrease this rebound effect.^[24,25]^

Potent BP treatment significantly reduced the relative risk of the main outcome of vertebral fracture incidence by 71% compared with the pooled comparators. Subgroup analysis indicated that the benefit was driven mainly by comparisons versus no therapy, where potent BP therapy reduced vertebral fracture risk by 78%. These findings are biologically feasible and in good accordance with the known pathophysiology of denosumab cessation. Previous observational studies, such as those by Anastasilakis et al and Tsourdi et al, showed a considerable rise in vertebral fracture rates after denosumab discontinuation in untreated patients, stressing the need for sequential therapy.^[26,27]^ Similarly, Everts-Graber et al reported decreased vertebral fracture rates in patients receiving zoledronic acid after discontinuation of denosumab compared with untreated controls.^[28]^ Our meta-analysis statistically synthesizes these results and consolidates the evidence for potent BP use following denosumab termination.

Interestingly, there was no statistically significant difference in vertebral fracture prevention with potent BPs versus SERMs. This finding may indicate that SERMs offer some antiresorptive protection after stopping denosumab, but the evidence is quite limited and imprecise.^[15,29]^ The limited sample sizes and large CIs in these subgroup analyses are more indicative of a lack of statistical power than true equivalence. Previous trials examining raloxifene following denosumab discontinuation have generated conflicting results, with some reports suggesting insufficient reduction of rebound bone turnover.^[30,31]^ Our results do not show superiority of potent BPs over SERMs, but should not be taken as proof of equal efficacy.

One of the most therapeutically important findings of this study was the large reduction in multiple vertebral fractures, with potent BP treatment linked with a 92% reduced risk overall and a remarkable 97% reduction compared with no treatment. One of the most impactful effects of denosumab cessation is the incidence of multiple vertebral fractures, which are linked with severe morbidity, discomfort, poor quality of life, and increased healthcare burden.^[32]^ Clusters of spontaneous multiple vertebral fractures occurring within months of denosumab cessation, especially in patients not on subsequent antiresorptive medication, have been reported in previous case studies and registry analyses.^[33,34]^ Our findings provide pooled evidence that potent BP medication is efficacious in reducing this severe rebound consequence. Moreover, the lack of variability in the no-therapy grouping indicates remarkable consistency across studies despite variation in study design and patient groups.

Potent BP medication resulted in a significant reduction in clinical vertebral fractures, with an 82% relative risk reduction. This conclusion is particularly noteworthy, since clinical vertebral fractures are symptomatic and clinically relevant events, not only radiological findings. It has been demonstrated in previous studies that vertebral fractures following discontinuation of denosumab are frequently consecutive and may be linked with large functional impairment. The consistency of our results across studies with low heterogeneity increases the confidence of this finding and further supports current guideline recommendations for sequential antiresorptive medication after discontinuing denosumab.^[22,29]^

In contrast, any fracture and non-vertebral fractures did not approach statistical significance, but pooled point estimates consistently favored potent BP medication. These results have several possible interpretations. Vertebral fracture is more closely connected with the rebound surge in bone turnover after withdrawal of denosumab, but non-vertebral fracture may be influenced by other factors such as falls, cortical bone quality, and patient comorbidities.^[35,36]^ Second, the limited number of included studies and events probably restricted statistical power for these outcomes. Third, the follow-up lengths were quite heterogeneous across studies, which may alter the rates of fracture capture. However, the same direction of benefit suggests that larger prospective trials may determine whether robust BPs may confer considerable protection against non-vertebral fractures.^[37,38]^

## 5. Strengths and limitations

A significant aspect of our study is the subgroup analysis of BP therapy versus no therapy and SERM-based methods. Previous reviews have tended to focus on BMD alterations or indicators of bone turnover rather than clinically relevant fracture outcomes. Previous evaluations were largely narrative and did not include pooled quantitative findings. Our meta-analysis offers more clinically relevant information on how to care for patients after denosumab by particularly comparing fracture incidence across different comparator regimens. Another distinguishing feature of this meta-analysis is the generally high methodological quality of the included studies. Most observational studies had strong NOS scores, and the single randomized trial indicated acceptable methodological rigor with “some concerns” on ROB 2 assessment. Moreover, most studies showed little heterogeneity, indicating the consistency of the results. The adoption of the GRADE system improves the transparency of evidence certainty.

However, multiple limitations must be overcome. First, a large portion of the included research was observational, which may include residual confounding and selection bias even in the presence of excellent methodological quality. Second, the evidence was not very certain due to only 1 randomized controlled trial being available. Third, several subgroup analyses, especially those comparing potent BPs with SERMs, had low sample sizes and broad confidence ranges. Fourth, clinical variability was high for the type, timing, and duration of BP therapy following termination of denosumab. Outcomes may have also been influenced by differences in the duration of past denosumab exposure and baseline fracture risk. Finally, the follow-up periods varied substantially between studies, which may influence the identification of long-term fracture events.

## 6. Future research and implications

Future research should emphasize the need for large multicenter randomized controlled trials comparing the different sequential medications after denosumab withdrawal, such as zoledronic acid, oral BPs, SERMs, and newer anabolic or dual-action medicines. Standardizing treatment timing following the final denosumab dose is particularly crucial, as delayed delivery may not sufficiently inhibit rebound bone turnover. Moreover, further investigations on bone turnover markers, BMD trajectories, timing of fractures, and long-term skeletal effects are needed to better delineate effective transition techniques. Future studies should also look at individualized techniques depending on the patient’s fracture risk, the duration of previous denosumab therapy, and comorbidity profiles.

## 7. Conclusion

This comprehensive review and meta-analysis indicate that potent BP therapy with ALN or zoledronic acid is correlated with a decreased risk of incident vertebral fractures, multiple vertebral fractures, and clinical vertebral fractures after denosumab discontinuation, particularly when compared with no subsequent therapy. Evidence comparing potent BPs with SERM therapy remains limited and inconclusive. No statistically significant reduction was observed for any fracture or non-vertebral fracture, likely because of sparse events and limited statistical power. Given the predominantly observational evidence base, further high-quality randomized and prospective comparative studies are needed to define the optimal sequential treatment strategy after denosumab discontinuation.

## Author contributions

**Conceptualization:** Asim Shah.

**Data curation:** Suleman Khan.

**Formal analysis:** Syeda Vilay Zehra Rizvi.

**Investigation:** Kanza Farhan.

**Resources:** Misbah Uddin.

**Methodology:** Syeda Umme Abiha Rizvi.

**Software:** Sabika Fatima.

**Visualization:** Ang Raj Karan.

**Validation:** Aizaz Anwar Khalid.

**Supervision:** Somaiya Ahmed.

**Writing – review & editing:** Ahmad Ali.




