# Supplementary material for: Potent bisphosphonate therapy for preventing fractures after denosumab discontinuation in osteoporosis: A GRADE-assessed systematic review and meta-analysis
Source: Medicine (Baltimore). 2026 Sep 4;105(36):e50542. doi: 10.1097/MD.0000000000050542 (PMC13549655; doi:10.1097/MD.0000000000050542)
Supplement: Supplementary file 1 [file medi-105-e50542-s001.docx]

**Supplementary Table S1: Search Strategy and literature search**

Pubmed (400)

(Osteoporosis OR bone loss OR low bone density OR skeletal fragility) AND (Denosumab OR anti-RANKL therapy OR RANK ligand inhibitor) AND (Diphosphonates OR bisphosphonate OR zoledronic acid OR alendronate OR risedronate OR ibandronate OR antiresorptive therapy OR bone resorption inhibitor) AND (Drug Discontinuation OR discontinuation OR withdrawal OR cessation OR stopping OR treatment interruption OR rebound effect) AND (Fractures OR fracture OR vertebral fracture OR osteoporotic fracture OR fragility fracture OR rebound fracture)

Cochrane (69)

((Osteoporosis OR bone loss OR low bone density OR skeletal fragility) AND (Denosumab OR anti-RANKL therapy OR RANK ligand inhibitor) AND (Diphosphonates OR bisphosphonate OR zoledronic acid OR alendronate OR risedronate OR ibandronate OR antiresorptive therapy OR bone resorption inhibitor) AND (Drug Discontinuation OR discontinuation OR withdrawal OR cessation OR stopping OR treatment interruption OR rebound effect) AND (Fractures OR fracture OR vertebral fracture OR osteoporotic fracture OR fragility fracture OR rebound fracture))

ClinicalTrials.gov (11)

((Osteoporosis OR bone loss OR low bone density OR skeletal fragility) AND (Denosumab OR anti-RANKL therapy OR RANK ligand inhibitor) AND (Diphosphonates OR bisphosphonate OR zoledronic acid OR alendronate OR risedronate OR ibandronate OR antiresorptive therapy OR bone resorption inhibitor) AND (Drug Discontinuation OR discontinuation OR withdrawal OR cessation OR stopping OR treatment interruption OR rebound effect) AND (Fractures OR fracture OR vertebral fracture OR osteoporotic fracture OR fragility fracture OR rebound fracture))

Scopus (246)
((Osteoporosis OR bone loss) AND (Denosumab) AND (Bisphosphonate OR zoledronic acid OR alendronate OR risedronate) AND (Discontinuation OR withdrawal OR cessation) AND (Fracture OR vertebral fracture OR osteoporotic fracture))
