# Supplementary material for: Potent bisphosphonate therapy for preventing fractures after denosumab discontinuation in osteoporosis: A GRADE-assessed systematic review and meta-analysis
Source: Medicine (Baltimore). 2026 Sep 4;105(36):e50542. doi: 10.1097/MD.0000000000050542 (PMC13549655; doi:10.1097/MD.0000000000050542)
Supplement: Supplementary file 2 [file medi-105-e50542-s002.docx]

| **Certainty assessment** | | | | | | | **Summary of findings** | | | | |
| --- | --- | --- | --- | --- | --- | --- | --- | --- | --- | --- | --- |
| **Participants (studies) Follow-up** | **Risk of bias** | **Inconsistency** | **Indirectness** | **Imprecision** | **Publication bias** | **Overall certainty of evidence** | **Study event rates (%)** | | **Relative effect (95% CI)** | **Anticipated absolute effects** | |
|  |  |  |  |  |  |  | **With Comparator** | **With Potent BP** |  | **Risk with Comparator** | **Risk difference with Potent BP** |
| **Incidence of vertebral fractures** | | | | | | | | | | | |
| 683 (5 non-randomised studies and 1 Randomized Controlled Trial) | not serious | not serious | not serious | not serious | Not assessable | ⨁⨁◯◯ Low | 16/149 (10.7%) | 17/534 (3.2%) | **RR 0.29** (0.14 to 0.59) | 16/149 (10.7%) | **76 fewer per 1,000** (from 92 fewer to 44 fewer) |
| **Incidence of Multiple vertebral fractures** | | | | | | | | | | | |
| 608 (4 non-randomised studies) | not serious | not serious | not serious | serious | Not assessable | ⨁◯◯◯ Very Low | 10/111 (9.0%) | 2/497 (0.4%) | **RR 0.08** (0.01 to 0.43) | 10/111 (9.0%) | **83 fewer per 1,000** (from 89 fewer to 51 fewer) |
| **Clinical Vertebral Fractures** | | | | | | | | | | | |
| 243 (3 non-randomised studies) | not serious | not serious | not serious | not serious | Not assessable | ⨁⨁◯◯ Low | 11/65 (16.9%) | 5/178 (2.8%) | **RR 0.18** (0.07 to 0.49) | 11/65 (16.9%) | **139 fewer per 1,000** (from 157 fewer to 86 fewer) |
| **Any fracture** | | | | | | | | | | | |
| 413 (3 non-randomised studies) | not serious | not serious | not serious | serious | Not assessable | ⨁◯◯◯ Very Low | 9/72 (12.5%) | 13/341 (3.8%) | **RR 0.35** (0.10 to 1.24) | 9/72 (12.5%) | **81 fewer per 1,000** (from 113 fewer to 30 more) |
| **Incidence of non-vertebral fractures** | | | | | | | | | | | |
| 221 (2 non-randomised studies) | not serious | not serious | not serious | serious^a^ | Not assessable | ⨁◯◯◯ Very low | 2/39 (5.1%) | 3/182 (1.6%) | **RR 0.43** (0.07 to 2.64) | 2/39 (5.1%) | **29 fewer per 1,000** (from 48 fewer to 84 more) |

**CI:** confidence interval; **RR:** risk ratio

.

**Supplementary Table S2: Grade Assessment**
